# High-resolution melting analysis reveals low *Plasmodium* parasitaemia infections among microscopically negative febrile patients in western Kenya

**DOI:** 10.1186/1475-2875-13-429

**Published:** 2014-11-15

**Authors:** Purity N Kipanga, David Omondi, Paul O Mireji, Patrick Sawa, Daniel K Masiga, Jandouwe Villinger

**Affiliations:** Martin Lüscher Emerging Infectious Diseases (ML-EID) Laboratory, Molecular Biology and Bioinformatics Unit, International Centre of Insect Physiology and Ecology (icipe), P.O. Box 30772, Nairobi, 00100 Kenya; St. Jude’s Clinic, International Centre of Insect Physiology and Ecology (icipe), P.O. Box 30, Mbita, 40305 Kenya; Department of Biochemistry and Molecular Biology, Egerton University Njoro Campus, P.O. Box 536, Egerton, 20115 Kenya; Department of Medical Biosciences, University of Western Cape, Private Bag X17, Bellville, 7535 Republic of South Africa; Yale School of Public Health, Yale University, 60 College Street, New Haven, CT 06510 USA

**Keywords:** Malaria misdiagnosis, antimalarial drug prescription, high resolution melting analysis (HRM), low-parasitaemia malaria detection, *Plasmodium* differentiation, febrile illness differential diagnostics

## Abstract

**Background:**

Microscopy and rapid diagnostic tests (RDTs) are common tools for diagnosing malaria, but are deficient in detecting low *Plasmodium* parasitaemia. A novel molecular diagnostic tool (nPCR-HRM) that combines the sensitivity and specificity of nested PCR (nPCR) and direct PCR-high resolution melting analysis (dPCR-HRM) was developed. To evaluate patterns of anti-malarial drug administration when no parasites are detected, nPCR-HRM was employed to screen blood samples for low parasitaemia from febrile patients without microscopically detectable *Plasmodium* infections in a rural malaria-endemic setting.

**Methods:**

Blood samples (n = 197) were collected in two islands of Lake Victoria, Kenya, from febrile patients without *Plasmodium* detectable by microscopy or RDTs. 18S rRNA gene sequences were amplified from extracted DNA by nPCR-HRM, nPCR, and dPCR-HRM to detect and differentiate *Plasmodium* parasites. The limits of detection (LoD) were compared using serial dilutions of the WHO International Standard for *P. falciparum* DNA. Data on administration of anti-malarials were collected to estimate prescription of anti-malarial drugs to patients with and without low parasitaemia *Plasmodium* infections.

**Results:**

The coupled nPCR-HRM assay detected *Plasmodium* parasites with greater sensitivity (LoD = 236 parasites/mL) than either nPCR (LoD = 4,700 parasites/mL) or dPCR-HRM (LoD = 1,490 parasites/mL). Moreover, nPCR-HRM detected and differentiated low-parasitaemia infections in significantly greater proportions of patients than did either nPCR or dPCR-HRM (p-value <0.001). Among these low-parasitaemia infections, 67.7% of patients were treated with anti-malarials, whereas 81.5% of patients not infected with *Plasmodium* parasites were treated with anti-malarials.

**Conclusions:**

The enhanced sensitivity of nPCR-HRM demonstrates limitations of differential febrile illness diagnostics in rural malaria endemic settings that confound epidemiological estimates of malaria, and lead to inadvertent misadministration of anti-malarial drugs. This is the first study that employs low-parasitaemia *Plasmodium* diagnostics to quantify the prescription of anti-malarial drugs to both non-malaria febrile patients and patients with low-parasitaemia *Plasmodium* infections. nPCR-HRM enhances low-parasitaemia malaria diagnosis and can potentially surmount the deficiencies of microscopy and RDT-based results in determining low-parasitaemia *Plasmodium* infection rates for evaluating malaria elimination efforts. The findings highlight the need for improved differential diagnostics of febrile illness in remote malaria endemic regions.

## Background

The World Health Organization (WHO) estimates that despite recent reductions in malaria incidence, malaria contributed to the morbidity and mortality of about 166 million and 564,000 people, respectively, in sub-Saharan Africa (SSA) in 2012 [1]. However, other epidemiological estimates nearly double these figures [2]. It is unclear how ‘presumptive diagnosis’ has influenced these statistics [3], since patients presenting with febrile illnesses with undetected parasitaemia by microscopy or rapid diagnostic tests (RDTs) are inadvertently integrated into the statistics, confounding prevalence measures of malaria in the process [4–6].

Appropriate diagnosis is not only important for ensuring prudent use of anti-malarial medications, and facilitating correct prognosis based on appropriate differential diagnosis [1, 7, 8], but also for tracking malaria elimination efforts [9–11]. Conventional microscopy [12] and RDT-based malaria diagnostics [13] used in malaria endemic regions of Africa have relatively low sensitivity and specificity in the detection of low-parasitaemia infections [14, 15]. Although the limits of detection (LoD) of parasitaemia by microscopy (100 parasites/μL or higher depending on technical skills) [16, 17] and RDT (50–400 parasites/μL) [18] may be well below the estimated parasite density threshold (3,500 parasites/μL) for initiation of febrile episodes [19], many clinicians in areas of high malaria endemicity are compelled to heuristically prescribe anti-malarials on the basis of clinical symptoms alone [6, 7, 20–22]. Such diagnostic and anti-malarial prescription practices in the absence of malaria positive microscopy and/or RDT results [6–8] may cause overlooking potentially severe non-malarial febrile illnesses that may coexist in malaria endemic tropical regions, such as bacterial, mycobacterial, fungal, and arthropod-borne viral infections [20, 11, 23, 24]. Indeed, even reliance on RDTs, which may also detect sub-clinical *Plasmodium* parasitaemia, has been implicated in undermining considerations of such differential diagnoses [10].

While low-parasitaemia *Plasmodium* diagnostics may be inappropriate for attributing febrile symptoms to malaria, effective malaria elimination efforts must be able to identify sub-clinical, low-parasitaemia *Plasmodium* infections that contribute to the infectious reservoir [12]. Molecular techniques are generally superior to antigen-detection tests and microscopy for detecting pathogens in biological samples [12, 13, 25, 26]. Specifically, techniques such as nested Polymerase chain reaction (nPCR) can enhance the sensitivity of detecting low-parasitaemia *Plasmodium* infections by increasing the total number of PCR cycles and by diluting the inhibitors present in the initial amplification reaction [27]. Similarly, direct PCR with high resolution melting (dPCR-HRM) analysis can distinguish between different *Plasmodium* species in a sample [28] and can hence provide better information on the relative abundance and composition of *Plasmodium* species in a study area.

This paper describes a novel molecular diagnostic technique that combines nested PCR with HRM analysis (nPCR-HRM) to detect and differentiate low-parasitaemia *Plasmodium* infections. The sensitivity of the novel coupled nPCR-HRM approach was compared to the performance of separate and independent implementations of nPCR [27] and dPCR-HRM [28] assays. The novel technique was further applied to quantify misadministration of anti-malarial drugs among patients presenting febrile symptoms without *Plasmodium* infections detectable by microscopy or RDTs in rural clinics on Rusinga and Mfangano Islands of Lake Victoria in Kenya.

## Methods

The study was approved by the National Ethical Review Board located at the Kenya Medical Research Institute, KEMRI (Approval Ref: Non-SSC Protocol #310). Project staff explained the study to the participants in the local language and provided participants with study information. Written informed consent was obtained from all patients sampled with febrile illness. Adults accompanying patients between 12 and 17 years old consented on behalf of the patients.

### Study sites

The study was conducted on the Lake Victoria islands of Mfangano (at Sena Health Centre, 0°28'24.10"S/34°4'2.54"E) and Rusinga (at Tom Mboya Health Centre, 0°24'0.66"S/34°9'50.52"E) located in western Kenya. Both islands have a population of between 20,000 and 30,000 people, and are in the Mbita constituency of Homa Bay County, a malaria endemic region in western Kenya. This region has recorded malaria prevalence as high as 50% between 2001 and 2004 [29]. Malaria transmission fluctuates with seasons, but is sustained throughout the year in the two islands. *Anopheles gambiae*, *Anopheles arabiensis*, and *Anopheles funestus* are the primary vectors of malaria [30] with *Plasmodium falciparum* as the most prevalent malaria parasite [31]. Rusinga Island is connected to the mainland via a 250-metre causeway constructed in the early 1980s through rock and earth filling. Mfangano Island is more isolated, located approximately 16 Km from the mainland. Both Sena and Tom Mboya Health Centres rely heavily on microscopy for malaria diagnostics, and use RDT’s inconsistently, as available. The area typically experiences long and short rainy seasons that are accompanied with an upsurge of malaria infections. The long rains extend from March to May and the short rains between August and December. Temperatures range from 17°C to 34°C with annual rainfall ranging between 700 mm to 1,200 mm [32].

### Human blood sampling

Blood sampled from patients visiting the two health centres presenting with febrile symptoms (fever above 37.5°C, headache, joint pains, diarrhoea, cough, abdominal pain, constipation, nausea) were screened for the presence of *Plasmodium* parasites by microscopy (thick blood smear) and RDT (CareStart™ Malaria HRP2(Pf)). Patients who provided samples with detectable *Plasmodium* parasites using these approaches were treated for malaria, allowed to go home and were excluded from this study. Blood (3 mL) from consecutive RDT and smear-negative febrile patients was drawn from consenting patients (12 years and above). All patients that had taken anti-malarial drugs within the preceding two weeks were excluded from the study. Blood samples from patients were collected using heparinized vacutainer collection tubes (MacMed Healthcare), aliquoted into three separate cryovials, and immediately stored in liquid nitrogen shippers. Trained hospital personnel also filled out structured questionnaires pertaining to demographic and clinical data of patients. To minimize selection bias, only two patients per week from each clinic were included in the study. Filled shippers and questionnaires were transported back to the laboratory for analysis. To protect patient anonymity, all blood samples and questionnaires were labelled with barcode identifiers. All samples were collected between May 2012 and October 2013, facilitated by staff in the Division of Disease Surveillance and Response (DDSR) of the Kenyan Ministry of Public Health and Sanitation.

### Extraction of total DNA from blood

Total DNA in blood samples were extracted by methods of Kawasaki [33] with minor modifications. Briefly, 50 μL of blood samples were separately transferred from cryovials to respective 1.5 mL eppendorf tubes containing 0.5 mL of Tris-EDTA (TE) buffer (10 mM Tris–HCl, 1 mM EDTA; pH 8.0). The samples were vortexed for ten seconds and centrifuged for five minutes at 13,000 relative centrifugal force (rcf) at 4°C. The resultant supernatants were discarded and the pellets were washed three times by re-suspending in the TE buffer, vortexing for ten seconds, and centrifuging for five minutes. The final pellets were re-suspended in 100 μL of proteinase K buffer (1.5 mM MgCl_2_, 50 mM KCl, 0.5% Tween 20, 100 μg/mL proteinase K and 10 mM Tris–HCl; pH 8.3), vortexed for ten seconds, and incubated at 55°C for an hour. The extracted DNA was incubated at 95°C for 10 minutes to inactivate the proteinase K, and then stored at −20°C until required.

### Detection of *Plasmodium*parasites by nPCR-HRM

The extracted DNA was amplified using nested PCR, targeting 18S rRNA genes in *Plasmodium* DNA. For the primary amplification step, forward (PL-1459-F: CTG GTT AAT TCC GAT AAC), and reverse (PL-1706-R: TAA ACT TCC TTG TGT TAG AC) primers were designed using Primer3 software [34]. The second pair of primers used for the nested amplification reaction were PL-1473-Forward (3’-TAA CGA ACG AGA TCT TAA-5’) and PL-1679-Reverse (3’-GTT CCT CTA AGA AGC TTT-5’) primers targeting a 204–270 base pair fragment (depending on the *Plasmodium* species) of a polymorphic region in the 18S rRNA gene [28]. Ten-fold dilutions of the primary PCR products were used as templates for the nested reactions. Each of the two amplification reactions were carried out in 10 μL final reaction volumes consisting of 1 μL DNA template, 2 μL Hot Firepol® HRM mix kit (Solis BioDyne, Estonia), 0.5 μL of 0.5 μM of both primers and 6 μL nuclease free PCR water. The touchdown PCR thermal conditions consisted of an initial denaturation at 95°C for 5 minutes, 45 cycles of denaturation at 94°C for 20 seconds, decreasing annealing temperatures from 65°C to 50°C for 25 seconds (cycles 1–5), 50°C for 40 seconds (cycles 6–10), 50°C for 50 seconds (cycles 11–45), and extension at 72°C for 30 seconds. A final extension of 72°C for 3 minutes was included before HRM analysis. Upon completion, the nested PCR process was transitioned into the melting phase (HRM) in the same closed tube system yielding distinct melting profiles in a Rotor-Gene Q® machine (QIAGEN, Germany). The set of conditions for HRM included 0.2°C incremental temperature increases from 75°C to 90°C, with fluorescence acquisition at the end of each 2 second temperature increment. DNA extracts from *P. falciparum* infected blood and deionized water were used as positive and negative controls, respectively. Products that produced curves distinct from the *P. falciparum* positive control were sequenced by Macrogen (Seoul, Korea) and aligned with reference sequences for *Plasmodium malariae* [GenBank:AB489193] and *Plasmodium ovale* [GenBank:AJ001527] using Geneious 6.1.6 software [35]. Protocols for nPCR [27] and dPCR-HRM [28] were also separately used on all samples for comparison purposes.

### Validation of nPCR-HRM sensitivity in comparison to established nPCR and dPCR-HRM protocols

To compare the detection limits of the three assays, we used the WHO International Standard for *P. falciparum* DNA, a standardized freeze-dried preparation of *P. falciparum* infected whole blood obtained from the National Institute for Biological Standards and Control (NIBSC; Hertfordshire, UK). Following NIBC recommendations, the lyophilized material was suspended in 500 μL of sterile, nuclease-free water to a final concentration of 1 × 10^9^ IU/mL, estimated to be 469,920 parasites/μL [36]. To establish and compare each assay’s LoD, we serially diluted the suspension in fresh uninfected whole blood. The sensitivity of each assay was tested using two replicate extractions of single-log (ten-fold) serial dilutions for the first four dilution points and four replicate extractions of each of the following eight half-log (3.16-fold) serial dilutions. The lowest concentration of DNA that tested positive in all replicates of a particular assay was set as the LoD. To narrow down the LoD further, additional tests were performed on half-log and two-fold dilutions of the lowest concentration that could be consistently detected among the original serial dilution series. To assess the rate of possible false positives due to cross-contamination during the nested amplification step of nPCR-HRM, 20 negative controls, including eight extraction negative controls, were distributed in every third position, such that each DNA standard serial dilution amplification was adjacent to a negative control reaction.

### Statistical methods

McNemar’s Chi-Square tests were used to compare the sensitivity of nPCR-HRM to nPCR and dPCR-HRM in detecting parasitaemia. Continuous/Gaussian data were compared using t-tests while Cohen’s Kappa was calculated to compare the agreement between the results obtained using the different methods. All statistical inferences were drawn on two-tailed distributions with α = 0.05. The data were analysed using R 3.0.1 software [37].

## Results

### Characteristics of study subjects

While 5,672 and 1,412 patients tested positive for malaria at Sena and Tom Mboya Health Centres, respectively, during the study period, 197 subjects with undiagnosed febrile illness were enrolled into the study. Subjects were between 12 and 70 years of age with a mean and median age of 31.9 and 28.0 years, respectively. There were 114 females and 81 males, with the gender of two individuals not reported. About 53.3% (n = 105) of the subjects were recruited from Sena Health Centre between Oct 15, 2012 and Oct 15, 2013 and the rest (n = 92) from Tom Mboya Health Centre between May 28, 2012 and Feb 18, 2013. Although 7,553 and 2,378 patients tested negative for malaria at Sena and Tom Mboya Health Centres, respectively, only two smear/RDT-negative patients from each clinic were included in the study every week to minimize time-based selection bias. The mean age for the subjects recruited from Sena Health Centre was 29.1 years old. This population was significantly younger than that recruited from Tom Mboya Health Centre, which averaged 35.4 years old (t = 3.3, df = 147.53, p = 0.001).

### Sensitivity comparison of the three techniques using serial dilutions of the WHO *Plasmodium*DNA International Standard

The LoD of the coupled nPCR-HRM technique was 236 parasites/mL, whereas the LODs of dPCR-HRM and nPCR were 1,490 parasites/mL and 4,700 parasites/mL, respectively.

### Malaria and *P. falciparum*prevalence as generated by the three techniques

**Figure 1:**
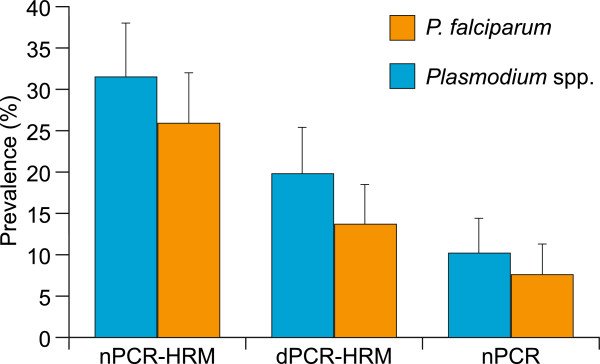
**Comparison of molecular techniques for low-parasitaemia** ***Plasmodium*** **detection among samples from microscopically negative febrile patients.** Total *Plasmodium* and *P. falciparum* prevalence among patient samples as determined by nPCR-HRM, dPCR-HRM, and nPCR. Error bars indicate the upper limit of the 95% CI.

Prevalence estimates of *Plasmodium* parasitaemia generated by our nPCR-HRM technique relative to those generated through nPCR or dPCR-HRM techniques are summarized in Figure 1. Results obtained by nPCR-HRM were in substantial agreement with those obtained by dPCR-HRM (Kappa = 0.699; 95% CI, 0.589-0.809) and fair agreement with those obtained by nPCR (Kappa = 0.395; 95% CI, 0.266-0.524). Nonetheless, our novel nPCR-HRM method detected significantly more cases of overall *Plasmodium* infections than did either dPCR-HRM (χ_1_^2^ = 21.04, p <0.001) or nPCR (χ_1_^2^ = 40.02, p <0.001). More specifically, nPCR-HRM also detected significantly more cases of *P. falciparum* infections than did either dPCR-HRM (χ_1_^2^ = 22.04, p <0.001) or nPCR (χ_1_^2^ = 34.02, p <0.001).

**Figure 2:**
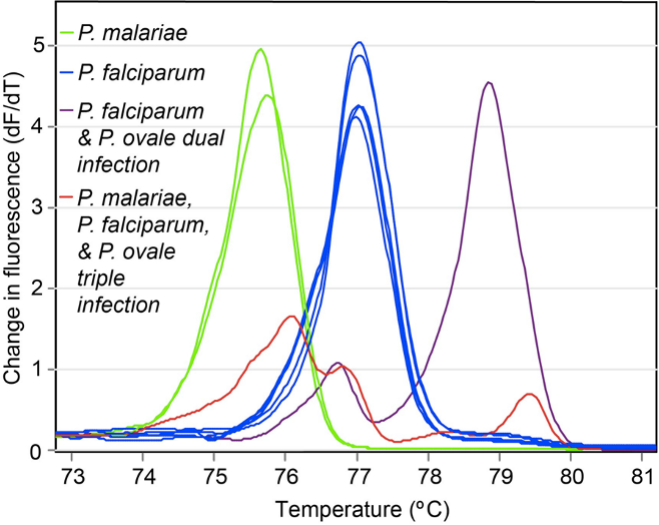
**Representative** ***Plasmodium*** **HRM profiles from a subset of the study samples.** Distinct melting profiles, represented by change in fluorescence units with increasing temperatures (df/dt) of patient samples with *P. malariae*, *P. falciparum*, and *P. ovale* infections.

Based on nPCR-HRM analyses, 62 (31.5%; 95% CI, 25.0-38.0) of the 197 subjects enrolled in the study had *Plasmodium* parasites in their samples, of which 53 (26.9%; 95% CI, 20.7-33.1) had *P. falciparum* parasites. While *P. falciparum* prevalence did not significantly differ between the two sampling sites during the study period (p = 0.137), the overall *Plasmodium* spp. prevalence was greater in samples collected at Tom Mboya Health Centre than those collected at Sena Health Centre (p = 0.013). Samples with *Plasmodium* collected at Tom Mboya Health Centre also had higher *Plasmodium* species diversity than those collected at Sena Health Centre. Samples collected at Tom Mboya Health Centre included 26 (72.2%) *P. falciparum* only infections, five (13.9%) *P. malariae* only infections, two (5.6%) *P. oval*e only infections, two (5.6%) double infections of *P. falciparum* and *P. oval*e, and one (2.8%) triple infection of *P. falciparum*, *P. malariae*, and *P. ovale*. Samples collected at Sena Health Centre had only two *P. ovale* infections with the remaining 24 infections being *P. falciparum*. The unique HRM profiles of the three *Plasmodium* parasites are represented in Figure 2. Based on dPCR-HRM, only 39 (19.8%; 95% CI, 14.2-25.4) of the patient samples had detectable *Plasmodium* parasites, among which 27 (13.7%; 95% CI, 8.9-18.5) were *P. falciparum* infections. nPCR revealed malaria parasites in only 20 (10.2%; 95% CI, 5.9-14.4) of the samples, among which 15 (7.6%; 95% CI, 3.9-11.3) were *P. falciparum* infections. No mixed infections were detected by either nPCR or dPCR-HRM. To verify that the nested amplification approaches did not yield false positives due to cross-contamination, these assays were repeated with the same samples in three successive runs, all of which produced identical results for the individual samples.

### Anti-malarial drug prescription patterns

**Figure 3:**
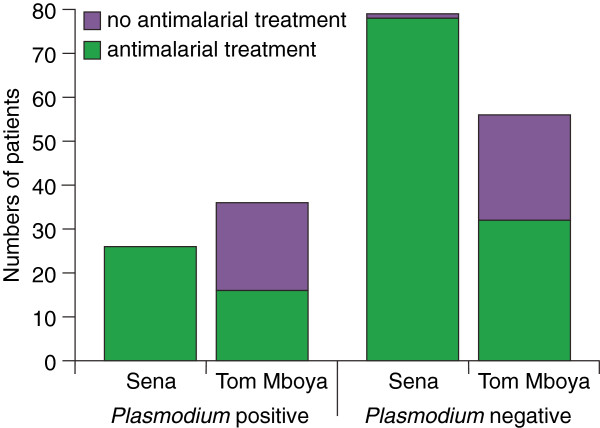
**Anti-malarial treatment of febrile patients with and without low parasitaemia** ***Plasmodium*** **infections identified by nPCR-HRM.** Numbers of patients treated or not treated with anti-malarial drugs in Sena and Tom Mboya Health Centres.

Among the 197 patients presenting with febrile illness enrolled in this study, 152 (77.2%) were treated with anti-malarial drugs. While 42 (67.7%) of the 62 patients with detectable *Plasmodium* parasites were treated with anti-malarial drugs, 110 (81.5%) of the 135 patients without detectable *Plasmodium* parasites were incorrectly treated with anti-malarial drugs. However, these patterns differed between the two Health Centres, as represented in Figure 3. While in Tom Mboya Health Centre, only 16 (44.4%) of the 36 patients with detectable *Plasmodium* parasites were treated with anti-malarial drugs, all 26 malaria patients with detectable parasites at Sena Health Centre were treated with anti-malarials. Yet, among the patients that did not have *Plasmodium* infection, more than half were treated with anti-malarial drugs at both Tom Mboya (32 out of 56, 57.1%) and Sena Health Centres (78 out of 79, 98.7%).

## Discussion

By combining the high sensitivity of nested PCR with real-time species differentiating HRM analysis, the reporting of *Plasmodium* infection prevalence that includes low-parasitaemia infections can be synergistically enhanced to improve size estimates of its infectious human reservoir in regions where malaria elimination efforts are being scaled up. Indeed, nPCR-HRM was able to detect *P. falciparum* at orders of magnitude lower concentrations than either nPCR or dPCR-HRM. The LoD of the coupled nPCR-HRM assay (236 parasites/mL) is however in the same range as TaqMan Probe assays validated using the same *P. falciparum* DNA standard (313 parasites/mL) [36]. Out of the 62 patient samples in which *Plasmodium* parasites were detected by the novel nPCR-HRM technique, the well-established nPCR [27] and dPCR-HRM [28] molecular techniques detected *Plasmodium* parasites in only 20 and 39 samples, respectively. The same is evident for *P. falciparum*, where nPCR-HRM identified 26.9% prevalence among microscopically negative febrile illness patients, while nPCR and dPCR-HRM identified only 10.2% and 13.7% prevalence, respectively. These results are consistent with the lower sensitivity of nPCR previously reported in comparison to dPCR-HRM [38], yet further demonstrate that nPCR-HRM is significantly more sensitive and specific than either nPCR or dPCR-HRM, and was also the only method able to detect double and triple infections with distinct *Plasmodium* parasites.

Although other novel molecular methods for *Plasmodium* detection have been reported recently, they either failed to undercut the detection thresholds of microscopy on clinical specimen [39] or were limited in ability to differentiate *Plasmodium* species [25, 36]. Due to these limitations in detecting and differentiating low-parasitaemia *Plasmodium* infections, no previous studies have assessed drug administration among subjects with malaria parasitaemia below the detection limits of standard microscopy and RDT.

The lack of diagnostic tools sufficiently sensitive to detect low *Plasmodium* parasitaemia in rural clinics in malaria endemic regions of SSA, and that can differentially diagnose other non-malaria febrile illnesses, including bacterial, mycobacterial, fungal, and arboviral infections, is a big challenge [12, 20–24]. While limited training of laboratory technicians has been suggested as a cause of poor malaria diagnoses [40], clinicians are left with little option, as a result of diagnostic limitations in rural clinics, but to treat patients with anti-malarials and antibiotics based on clinical symptoms [6, 7, 20–22].

Febrile illness misdiagnosis can be significant in malaria endemic settings, resulting in a myriad of downstream issues including improper treatment, chronic suffering of patients as the underlying cause of illness remains unknown, and drug wastage [7]. Moreover, poor malaria diagnostics can undermine the diagnosis of non-malarial febrile illnesses [10, 23, 24]. Due to the limitations of microscopy and RDTs in detecting low-parasitaemia malaria [14, 15] and clinical heuristic treatment practices [6, 7, 20–22], 197 febrile patients from both study sites were diagnosed as not having malaria, but were still given different medications on the basis of clinical signs. In endemic regions with high transmission intensity, such presumptive fever management may be justifiable, as test-based management may not be safe or cost effective [10]. Because clinicians are limited to heuristic methods of clinical diagnoses in such cases, treatment patterns are likely to differ between clinics and clinicians. Indeed, while all low *Plasmodium* parasitaemia cases at Sena Health Centre were treated with anti-malarial drugs, only 44.4% of low-parasitaemia patients at Tom Mboya Health Centre were treated with anti-malarial drugs. However, in both Health Centres, the majority of anti-malarial drugs were prescribed to patients in whom we did not detect *Plasmodium* parasitaemia.

The discrepant prescribing patterns between the two study sites may be due to the over three times greater numbers of patients with microscopically positive malaria observed at Sena Health Centre than at Tom Mboya Health Centre during the respective sampling periods. Accessibility to other health facilities may also have influenced prescribing practices. Compared to Tom Mboya Health Centre on Rusinga Island, Sena Health Centre on Mfangano Island is more isolated from the mainland where patients access other nearby and specialized health centres that they may be referred to for assessment of differential diagnoses. Due to the relatively limited accessibility to such referral clinics for patients on Mfangano Island, clinicians may have had a stronger tendency to prescribe the more readily available anti-malarials at Sena Health Centre.

Individuals with extremely low-parasitaemia *Plasmodium* infection are likely to have endemic stability to malaria infection and may be able to tolerate a low degree of parasitaemia with absence of clinical disease [19]. Therefore, anti-malarial drugs do not treat febrile illness patients with sub-microscopic *Plasmodium* infections for the underlying differential diagnosis of their febrile symptoms [10]. Nonetheless, treatment of patients with sub-microscopic *Plasmodium* infection, may contribute to integrated malaria elimination/control efforts by reducing the size of the infectious reservoir within communities [9–11].

This study demonstrates how improper febrile illness diagnoses confound prevalence estimates of *Plasmodium* infections and other differential diagnoses. Some non-malaria febrile patients are diagnosed and treated for malaria based on their clinical symptoms, and may be inadvertently integrated into epidemiological statistics [3–6], while some low-parasitaemia *Plasmodium* infections go unrecognized [2]. As a result, malaria burden and infection estimates suffer from unknown large margins of error [4]. The complementary use of sensitive and specific molecular based methods informs size estimates of the potential infectious reservoir of malaria in endemic regions, while also providing estimates of undifferentiated febrile illness burden.

Improved understanding of *Plasmodium* infection rates will translate into the development of better rural malaria diagnostics and treatments, ultimately reducing gametocyte incidence rates in asymptomatic populations with low-parasitaemia malaria [12]. While individuals with submicroscopic parasitaemia have significantly lower *Plasmodium* transmission rates to mosquito vectors [41, 42], they may still constitute a considerable proportion of the human infectious reservoir [12, 42, 43], a phenomenon that probably prevails among individuals with endemic stability to malaria infection in the endemic study sites. Therefore, more accurate parasite-detection based diagnosis and treatment may reduce malaria transmission [42]. Low-parasitaemia malaria can result in acute febrile illness [5] and individuals that may have low-parasitaemia in the peripheral blood may still require anti-malarial treatment due to higher parasite loads that can still be sequestered in capillaries [44]. In contrast, acute febrile illnesses have many aetiologies ranging from bacterial, fungal and mycobacterial infections to arboviral infections [24]. These unknowns complicate appropriate febrile illness diagnosis and treatment. Clear guidelines and policies on the management of non-malaria acute febrile illnesses are required urgently, particularly in malaria endemic regions.

This study’s approach to low-parasitaemia *Plasmodium* detection and species differentiation is a valuable molecular tool that can be used to complement microscopy and RDT and enhance understanding of specific *Plasmodium* infection rates. While sensitive and specific, nPCR-HRM was not designed as a diagnostic tool for health clinics, as it requires real-time PCR infrastructure, specialized training to avoid the cross-contamination risks associated with nested PCR assays, and more man power in comparison to RDTs. Recent developments in both loop-mediated isothermal amplification (LAMP) [25] and isothermal lateral flow-recombinant polymerase amplification (LF-RPA) [26] assays show promise as true point-of-care tests with high specificity and sensitivity for detecting low-parasitaemia *Plasmodium* infections. Nonetheless, nPCR-HRM can be employed for monitoring low-parasitaemia infection rates within malaria endemic regions to assess the progress of regional malaria elimination efforts.

## Conclusions

This is the first study that employs low-parasitaemia *Plasmodium* diagnostics to quantify both the over-prescription of anti-malarial drugs in non-malaria febrile patients and under-prescription of anti-malarial drugs in low-parasitaemia malaria patients. While patient febrile illness symptoms cannot be attributed to any of the sub-microscopic *Plasmodium* infections identified in this study, consistent anti-malarial treatment limited to such cases should contribute to regional malaria elimination efforts [9–11]. Other recent cohort studies have contrasted presumptive clinical diagnoses and anti-malarial drug treatments with malaria parasitaemia detectable by microscopy [21, 23, 24], as microscopy [8], alongside RDTs [5], is the mainstay of present malaria diagnostic practices in most malaria endemic regions of Africa. In a low transmission area clinical cohort study in Tanzania, Reyburn and colleagues found that 38% of suspected malaria patients were treated for malaria without further testing and 48% of microscopically malaria negative febrile patients were presumptively treated with anti-malarial drugs [21]. More recently, Crump and colleagues showed that only 2.7% of patients diagnosed with malaria in two hospitals in northern Tanzania were actually malaria positive as determined by microscopy [24]. Considering *Plasmodium* infection prevalence, we further demonstrate the inadequacy of rural diagnostics in detecting low-parasitaemia infections.

While patients with undiagnosed and untreated low-parasitaemia *Plasmodium* infections may not suffer from acute febrile illness as a result of malaria, they may contribute to a significant proportion of the infectious reservoir during their asymptomatic state. Conversely, the lack of diagnostics and treatment for unrecognized differential febrile illness diagnoses may have severe health implications that cannot be addressed properly. Epidemiological estimates need to take into account the considerable proportions of asymptomatic low-parasitaemia *Plasmodium* infections that contribute to the infectious reservoir, while also considering the health impacts of unrecognized differential diagnoses of febrile illness in malaria endemic regions.

## Abbreviations

PCR: Polymerase chain reaction
HRM: High resolution melting
nPCR: Nested Polymerase chain reaction
nPCR-HRM: nested Polymerase chain reaction – high resolution melting
dPCR-HRM: Direct Polymerase chain reaction – high resolution melting
RDT: Rapid diagnostic test
WHO: World Health Organization
SSA: sub-Saharan Africa
KEMRI: Kenya Medical Research Institute
DDSR: Division of Disease Surveillance and Response
CI: Confidence Interval
LAMP: Loop-mediated isothermal amplification
LF-RPA: Lateral flow-recombinant polymerase amplification
LoD: Limits of detection.
